# The asexual cycle of apicomplexan parasites: new findings that raise new questions

**DOI:** 10.1016/j.mib.2013.07.017

**Published:** 2013-08

**Authors:** Markus Meissner

**Affiliations:** Wellcome Trust Centre for Molecular Parasitology, III MVLS Sir Graeme Davies Building, University of Glasgow, Glasgow, UK

**Current Opinion in Microbiology** 2013, **16**:421–423

For a complete overview see the Issue

Available online 8th August 2013

1369-5274/$ – see front matter, © 2013 Elsevier Ltd. All rights reserved.

**http://dx.doi.org/10.1016/j.mib.2013.07.017**

The phylum apicomplexa consists of more than 5000 species, including important human and veterinary pathogens that have a huge, global effect on human and animal health. While *Plasmodium* spp. (the causative agent of malaria) is the most notorious member of this phylum causing approximately 660 000 deaths a year, other apicomplexans are among the most relevant foodborne pathogens of global socio-economic relevance. For example *Eimeria* spp. are the most important protozoan pathogens of poultry. Other important apicomplexan pathogens include *Cryptosporidium* spp., *Neospora caninum*, *Theileria* spp. or *Toxoplasma gondii*. While most apicomplexans show a relatively small host range, *Toxoplasma gondii*, infects all nucleated cells from warm blooded animals and has a huge impact on both human and animal health. This parasite is a major cause of abortion in livestock, with significant economic losses to sheep and goat breeders. In addition, it is one of the leading causes of death attributed to foodborne illness. Especially the primary infection during pregnancy, which can cause serious damage of the unborn child and the reactivation of dormant tissue cysts in immunocompromised patients are the most common complications caused by a *Toxoplasma* infection.

Apicomplexan parasites are often considered as unconventional eukaryotes due to several adaptations that were acquired during the evolution of an intracellular life style that involves transmission in between different hosts. The asexual life cycle, although at first site relatively simple, can be summarised as periodic events of invasion, replication and host cell egress. In order to successfully complete this lytic cycle apicomplexans evolved several unique organelles, pathways and structural features. For example micronemes and rhoptries are unique secretory organelles essential for host cell invasion and egress, the apicoplast is a remnant plastid that fulfils important metabolic roles during the intracellular stage and parasite proteins are transported into the host cell via a translocon. These effectors modify and reorganise the host cell as impressively seen in case of infected erythrocytes by *P. falciparum.* Modern reverse and forward genetic technologies that have been established and optimised in these parasites were indispensable to establish and verify the roles of essential factors and pathways during these processes. This 6-article section gives a perspective of where we stand today, summarising the current view of how apicomplexans evolved unique secretory organelles, regulate their secretion and use effector proteins for host cell invasion, host cell modulation and host cell egress. Furthermore, unique metabolic pathways are discussed that are essential during the intracellular life stage. This issue also highlights some of the differences between the two best studied model systems for apicomplexan parasites, *Plasmodium* and *Toxoplasma.* While very similar at first sight, these two parasites cannot be directly compared when it comes to fundamental aspects during the asexual life cycle. Although some of the mechanisms might be conserved, several unique pathways and mechanisms exist. Therefore, one has to consider the differences rather than the similarities of these parasites, which are also determined by the co-evolution with their respective hosts.

The basic cell biology of apicomplexan parasites has puzzled researchers from early ultrastructural analysis. These parasites possess a highly reduced secretory system, consisting only of a single Golgi stack. Yet, this system is highly polarised with unique secretory organelles anchored at the apical tip of the parasite. In the first article by Klinger *et al.*, the evolutionary history of this organisation is compared with other protozoans. Most of our knowledge on cell biology comes from studies on ophistokonts (from yeast to human). Given the huge evolutionary distance, conserved proteins can fulfil different roles. In this review the cell biology and evolutionary history of apicoplast, inner membrane complex (IMC) and secretory organelles is discussed and a hypotheses is proposed that suggests a significant re-organisation of the endolysosomal system in apicomplexans.

In the second article by Sharma and Chitnis the highly complex regulation of key effector molecules during host cell invasion is reviewed. Apicomplexan parasites invade the host cell in a highly coordinated manner that involves the step wise secretion of micronemes and rhoptries that culminate in the formation of a tight junction (TJ) through which the parasite invades the host cell in an active process. This co-ordination involves a complex interplay of signalling molecules and second messengers, such as Calcium-signalling or cAMP that are known to be involved in microneme release. As the authors note regulation of rhoptry release is still enigmatic. Although parasite ligands that are involved in this process have been identified, the signalling cascades regulating this step are unknown. Furthermore the authors speculate how external signals lead to activation of cAMP and Ca^2+^ dependent pathways to regulate secretion and the parasite motor that is implicated in host cell invasion. The authors conclude that a global study of post-translational modifications is involved in the regulation of the complex invasion machinery.

In the following article Meissner *et al.*, point out that recent reverse genetic data indicate that the current model for host cell invasion might need to be revised, since parasites devoid of actin and myosin A, the core motor for gliding motility and the main ligand MIC2 are still capable of host cell invasion. The authors discuss these results in the context of the actual literature and point out that previous data only suggest that the current hypothesis is correct. However, this hypothesis is based on contradictory inhibitor studies and knockdown mutants for critical invasion factors that in no case led to the expected block in host cell invasion, leading to the conclusion that apicomplexans have other mechanisms at their disposal that allow them entry into the host cell. Furthermore, it is pointed out that other apicomplexans, such as *Theileria* or *Cryptosporidium* are well capable of invading the host cell using “alternative” mechanisms.

Once inside the host cell apicomplexans “renovate” their new home and secrete a huge amount of proteins into their new home to modulate host cell responses and to change the structural interior of the host cell. This requires the secretion of a large repertoire of proteins that need to be transported across multiple membranes. While it is still enigmatic how constitutively secreted proteins reach the host cell in the case of *T.gondii*, in the case of *Plasmodium* most secreted proteins contain an export element (PEXEL/HT). However, recent work also identified a growing number of PEXEL negative proteins (PNEPs) adding to the complexity of the malaria secretome. Marti and Spielmann discuss in their review the current findings on the complex nature of the *Plasmodium* export pathway and the role of the enigmatic translocon complex that is believed to facilitate the transport of proteins into the cytosol of the host cell.

During the intracellular life stage, apicomplexans rely on the hosts metabolic pathways and energy production. Yet these parasites retained endosymbiotic organelles – mitochondria and a plastid like organelle, the apicoplast. Sheiner *et al.*, highlight in their contribution the complex evolutionary interplay of these organelles that is essential for the synthesis of important metabolites that cannot be scavenged from the host cell. The authors highlight and discuss common themes, but also differences between *Plasmodium* and *Toxoplasma.*

In the final review of this issue, Carruthers and Blackman summarise our current knowledge on processes that are important for *Plasmodium* and *Toxoplasma* egress from the host cell*.* The authors point out that despite immense efforts by several groups, many events during egress are still unknown. For example the natural triggers for egress in *Plasmodium* blood stages are not yet identified. The authors also discuss that although at first sight the events leading to egress appear similar between *Toxoplasma* and *Plasmodium*, the basic molecular machinery appears to be diverse and both parasites use different effector molecules during egress. It remains to be seen if different apicomplexans use distinct egress mechanisms, possibly determined by the different host cell environment they inhabit.

These short reviews are intended to give a general overview into some of the questions and hot topics discussed in relation to the asexual cycle of apicomplexan parasites. They however only provide a glimpse into what makes this research field so attractive and how research on these unconventional eukaryotes can be so fascinating. By finding answers to some of these questions we will not only expand our general, basic knowledge, it will hopefully also lead to new avenues for intervention strategies in our battle against these important human and veterinary pathogens.

